# A Spatially Explicit Dual-Isotope Approach to Map Regions of Plant-Plant Interaction after Exotic Plant Invasion

**DOI:** 10.1371/journal.pone.0159403

**Published:** 2016-07-27

**Authors:** Christine Hellmann, Christiane Werner, Jens Oldeland

**Affiliations:** 1 Experimental and Systems Ecology, University of Bielefeld, Bielefeld, Germany; 2 Ecosystem Physiology, University of Freiburg, Freiburg im Breisgau, Germany; 3 Biodiversity, Evolution and Ecology of Plants, Biocentre Klein Flottbek and Botanical Garden, University of Hamburg, Hamburg, Germany; Estacion Experimental de Zonas Aridas - CSIC, SPAIN

## Abstract

Understanding interactions between native and invasive plant species in field settings and quantifying the impact of invaders in heterogeneous native ecosystems requires resolving the spatial scale on which these processes take place. Therefore, functional tracers are needed that enable resolving the alterations induced by exotic plant invasion in contrast to natural variation in a spatially explicit way. ^15^N isoscapes, i.e., spatially referenced representations of stable nitrogen isotopic signatures, have recently provided such a tracer. However, different processes, e.g. water, nitrogen or carbon cycles, may be affected at different spatial scales. Thus multi-isotope studies, by using different functional tracers, can potentially return a more integrated picture of invader impact. This is particularly true when isoscapes are submitted to statistical methods suitable to find homogeneous subgroups in multivariate data such as cluster analysis. Here, we used model-based clustering of spatially explicit foliar δ^15^N and δ^13^C isoscapes together with N concentration of a native indicator species, *Corema album*, to map regions of influence in a Portuguese dune ecosystem invaded by the N_2_-fixing *Acacia longifolia*. Cluster analysis identified regions with pronounced alterations in N budget and water use efficiency in the native species, with a more than twofold increase in foliar N, and δ^13^C and δ^15^N enrichment of up to 2‰ and 8‰ closer to the invader, respectively. Furthermore, clusters of multiple functional tracers indicated a spatial shift from facilitation through N addition in the proximity of the invader to competition for resources other than N in close contact. Finding homogeneous subgroups in multi-isotope data by means of model-based cluster analysis provided an effective tool for detecting spatial structure in processes affecting plant physiology and performance. The proposed method can give an objective measure of the spatial extent of influence of plant-plant interactions, thus improving our understanding of spatial pattern and interactions in plant communities.

## Introduction

Understanding the processes that determine spatial patterns in field data is a major goal in plant ecology [1]. One approach to analyze spatial patterns is to group spatially explicit measurements into separable homogeneous clusters, aiming at identifying biotic and abiotic factors that explain the variation between the clusters. Spatial clustering is a suitable method which is commonly applied to large spatial datasets, such as ecoregions [2,3] or datasets in epidemiology [4–6]. However, it is seldom applied to study the spatial context of functional processes within plant communities. For example, the spatial extent in which individual plants interact with one another or with the abiotic environment is often unknown [7]. One case where the area influenced by individual plants is of special interest is the invasion of exotic plant species into well-established native plant communities.

Invasive species are known to affect native ecosystem functioning in numerous ways and have been recognized as one of the major drivers of biodiversity loss [8]. Plant invaders have been shown to directly alter community composition and structure, as well as to change the native environment by modifying biogeochemical cycles, notably carbon, water and nutrient cycling [9–12]. However, although it is a major objective to measure the impact of invasive species on plant communities [13,14], spatially explicit approaches in invasion ecology mostly consider large scale distribution patterns (see e.g. [15]), while approaches to quantify the local area of impact are rare.

Stable isotopes can provide valuable tracers for several of the processes affected by invasion at local scales. δ^15^N has been proposed as an integrator of N cycling [16–18] and can be used to trace N sources of plants [19,20], such as N introduced to the native system by an invasive species [21,22]. Foliar δ^13^C is related to photosynthetic ^13^C-fractionation, which depends on the ratio of internal to external CO_2_ partial pressure, and can thus add additional information on changes in intrinsic water use efficiency (WUE_i_) of C_3_ species [23], which has been used in many studies (e.g. [24,25]).

Spatially explicit representations of stable isotope values, i.e. isoscapes [26], allow for resolving and analyzing variations in functional tracers on multiple spatial scales. For example, δ^15^N isoscapes have been successfully used to quantify the impact of an N_2_-fixing invasive *Acacia spp*. [22]. However, recent work shows that the area influenced by invasion differs depending on the tracer used, i.e. multiple tracers may detect different interaction ranges with respect to the observable processes. Hence, instead of analyzing each tracer individually, a method that includes all available tracers or isoscapes would provide a more complete picture. Such a multivariate isoscape would combine the information contributed by different tracers to characterize the region influenced by invasion, integrating the diverse processes involved and enabling to identify subregions of invader influence.

A well-established group of unsupervised methods to find subgroups in multivariate data is cluster analysis [27]. Clustering of georeferenced isotopic values (isoscapes) has successfully been used for example to track animal migration on a continental scale [28,29], to understand trophic interactions [30], and to determine origins of biological materials on country level (e.g. [31]). However, clustering of isoscapes has mainly been applied within contexts of zoology and/ or geographic assignment while no examples with plant ecological scope are known to us from the literature. Furthermore, the mentioned studies were always conducted on large spatial scales, i.e. on country or continental scale, while studies based on e.g. individual plants in an area of less than a hectare do not exist, yet are possible.

The aim of this study was to evaluate the suitability of spatial clustering to find subregions within plant communities with different mechanisms and/ or intensity of plant-plant interactions. For this purpose, multiple raster layers representing spatially explicit measurements of informative functional tracers, i.e., isoscapes were used. Specifically, we aim to identify different regions of influence in a Portuguese dune ecosystem invaded by the N_2_-fixing *Acacia longifolia* (Andrews) Willd., a highly problematic invasive species in Mediterranean regions, using δ^15^N and δ^13^C isoscapes of a native indicator species (*Corema album* L.) combined with information on foliar N concentration. It has already been proven that ^15^N isoscapes represent an effective spatial tracer for the impact of *A*. *longifolia* [21,22]. Using a multivariate approach and including δ^13^C will enable to reveal additional facets of plant-plant interactions that may also differ in their spatial extent. For example, while foliar δ^15^N can be used to trace N input from N_2_-fixation by the invasive species, this may have positive or negative impacts on water use efficiency, depending on weather facilitative or competitive processes dominate the interaction. Thus, including an additional tracer will enable to distinguish between different types of interactions occurring in the community. We seek to detect homogeneous regions in the multivariate data in an objective, unsupervised way, that is, with no previous assumptions on data distribution and grouping factors used in the analysis. This will be the first study using a multivariate isoscape clustering approach for disentangling plant-plant interactions on the plant community level in the context of invasion biology.

## Materials and Methods

### Study site

The field site is located in a sand dune ecosystem near Pinheiro da Cruz at the Atlantic coast of Portugal (38°15.4’N, 8°46.3’ W) and was accessed with permission of the Estabelecimento Prisional de Pinheiro da Cruz. Climate is Mediterranean with Atlantic influence, with mean annual temperature of 16.6°C and mean annual precipitation of 735 mm for the nearest meteorological station in Setúbal, 38°33’N, 08°53’W, in the period of 1981–2010 [32]. Summers are dry with high temperatures and precipitation is mostly concentrated in the winter month. Soils are arenosols (FAO classification) with poor water retention capacity [33], low organic matter content and low N and P availability [21]. The native vegetation forms a sparse canopy with large proportions of bare sand. Dominant native species are the dwarf shrubs *Corema album* L. (Ericaceae), the N_2_-fixing *Stauracanthus spectabilis* Webb (Fabaceae), and numerous young as well as occasional adult pine trees (*Pinus pinaster* Aiton, Pinaceae). The area is invaded by the exotic N_2_-fixing *Acacia longifolia* (Andrews) Willd. (Fabaceae), a shrub or small tree of ca. 1–8 m height which forms several dense, monotypic stands in the studied ecosystem. These stands differ from the native vegetation in terms of structure and plant diversity and previous studies have shown that the ecosystem gets enriched with nitrogen in the surrounding of *A*. *longifolia* [21,22,34].

### Foliage sampling

Three plots of 1,000 m^2^ area (20 x 50 m) were established in the dune system and subdivided into 2 x 5 m subplots. In the three plots, *C*. *album* occurred frequently with even spatial coverage, allowing for a well-resolved spatial sampling design. Plot 1 and 2 each comprised stands of the invasive *A*. *longifolia*, with 17% and 7% of the plot area covered by the invader, respectively. These plots were sampled in November 2009 [22]. Additionally, a control plot (plot 3) of uninvaded native vegetation was established in April 2011. For plots 1 and 2, foliage of *C*. *album* was collected from each plant and samples were pooled per subplot. For plot 3, one random individual of *C*. *album* was sampled per subplot and the exact location of the individual was recorded. Regarding sampling strategies, we confirmed that data of individual plants sampled along a gradient corresponded well to interpolated data obtained from pooled subplots. Only current year, fully expanded, sunlit foliage was sampled on all occasions. Comparability between seasons was confirmed by comparing N concentration, δ^15^N and δ^13^C of plot 1 from spring 2008 with resampled data from November 2009, which showed no significant differences in δ^15^N and δ^13^C (Mann-Whitney-U test, W = 1171, *p* = 0.14 and W = 1362, *p* = 0.79) and only marginal deviations in N concentration, with values being slightly higher in May, showing an average enrichment of 1 g N*kg^-1^ (W = 1037, *p* = 0.02).

### Analysis of isotopes and N concentration

Leaf samples were dried at 65°C for at least 48 h and then homogenized and ground to a fine powder using a ball mill (Retsch, Haan, Germany). Samples were analyzed for N concentration and C and N isotopic composition in an elemental analyzer (HEKAtech GmbH, Weinberg, Germany) coupled to a continuous flow stable isotope ratio mass spectrometer (ISOPRIME, Elementar, Hanau, Germany) at the University of Bielefeld, Germany. All samples were measured against a laboratory standard (IVA33802156, IVA Analysetechnik e.K., Meerbusch, Germany) and international standards (IAEA-NO-3 for plot 1 and 2 δ^15^N and IAEA-N-1 for plot 3 δ^15^N, International Atomic Energy Agency, Vienna, Austria). Repeated measurement precision was ≤0.5 g N*kg^-1^ for N concentration, ≤0.2‰ for δ^15^N and ≤0.1‰ for δ^13^C. Isotopic composition is reported in delta-notation referenced to the international IAEA standards (air N_2_ for δ^15^N and V-PDB for δ^13^C).

### Isoscapes

Geographic positions of the plot corners were recorded and *C*. *album* distribution and *A*. *longifolia* canopies were digitized based on hand-drawn maps in QGIS 2.4 [35]. Foliar N concentration and δ^15^N of plot 1 and 2 were re-analyzed from [22]. δ^13^C was measured from the same samples of [22]. Additionally, an uninvaded control (plot 3) was sampled for both δ^13^C and δ^15^N. Foliar N concentration, δ^15^N and δ^13^C of *C*. *album* in the three plots were interpolated to continuous surfaces using variogram fit and ordinary kriging in R 3.2.1 [36] with the package gstat [37]. For variograms, cutoff distance was set to the default (1/3 of the longest diagonal = 17.95 m). In the variogram fits, all models proposed by the function *vgm* were tested with different lag distances, including five uniform (1 m, 1.2 m (default), 2 m, 3 m, 4 m) and one non-uniform segmentation (with the following boundaries: 1, 2, 3, 4, 5, 6, 7, 9, 12, 15, 18 m). The best combination of lag-distance and variogram model was chosen by means of a leave-one-out cross-validation procedure using the function *krige*.*cv*. For this purpose, the variogram fitting and the kriging was done repeatedly and each raster cell of the plot was left out from the process once and predicted from the resulting model in this run. The model that yielded the smallest root mean square error (RMSE) for predicted values and the highest R^2^ for the regression of predicted on measured values was chosen as the final model. For plots 1 and 2, additionally, the distance of each raster cell to the closest cell occupied by *A*. *longifolia* was calculated (R package raster, [38]).

### Cluster analysis

To test whether the raster cells of the three plots would fall into discernable clusters based on their N concentration, δ^15^N and δ^13^C values, a model-based cluster analysis [39] was performed using the package mclust [40] in R. 3.2.1 [36]. More conventional cluster algorithms like hierarchical clustering and *k*-mean partitioning approaches are usually distance-based and may be unreliable when clusters are not spherical, i.e., variable in volume, shape and/or orientation [41]. Furthermore, it is often difficult to decide on the correct number of clusters, particularly in the presence of overlapping groups. Model-based clustering assumes that the data consist of a number of subgroups (the clusters), in each of which the variables have a different multivariate probability density function [39,40]. Model-based cluster analysis yields an estimation of the parameters of these probability density functions and calculates the posterior probabilities of cluster membership for each sample. The optimal number of clusters can then be chosen by employing a model selection criterion. The function *Mclust* uses the expectation-maximization (EM) algorithm for normal mixture models with a variety of covariance structures [39]. Ten models were tested with different combinations of the following constraints: spherical, diagonal or ellipsoidal distribution; equal or varying volume; equal or varying shape; equal or varying orientation [40]. Model selection was achieved by maximizing the Bayesian Information Criterion (BIC, [42]). For the three plots in our study, BIC plotted against the number of clusters increased strongly with the first four to six clusters and then levelled off and reached a plateau for some 10–20 additional clusters before decreasing (S1 Fig). Abiding by the rules of parsimony, the lowest number of clusters at the beginning of the plateau was chosen.

For the interpretation of the clusters, we produced boxplots for each model parameter, cluster and plot combination. A Kruskal-Wallis rank sum test followed by a post-hoc multiple comparison test correcting for α-inflation (function *kruskalmc* from the R package pgirmess, [43]) was used to test for significant differences between cluster medians of N concentration, δ^13^C and δ^15^N. For a better interpretation of the spatial configuration of clusters in relation to the invader, we also plotted the clusters against the median distance to the closest *A*. *longifolia* canopy within the plots.

In order to be able to compare clusters across the three plots, we used the median values of N concentration, δ^15^N and δ^13^C from the clusters retrieved by *Mclust* in a hierarchical cluster analysis (unweighted pair group method with arithmetic mean (UPGMA) with Euclidean distance, see [41] for detailed information on algorithm and distance measure) using the function *agnes* of the package cluster [44]. Euclidean distance was used as all data were on the same scale. The optimal number of clusters was defined as the clustering solution with the highest Silhouette value [45].

## Results

**Fig 1:**
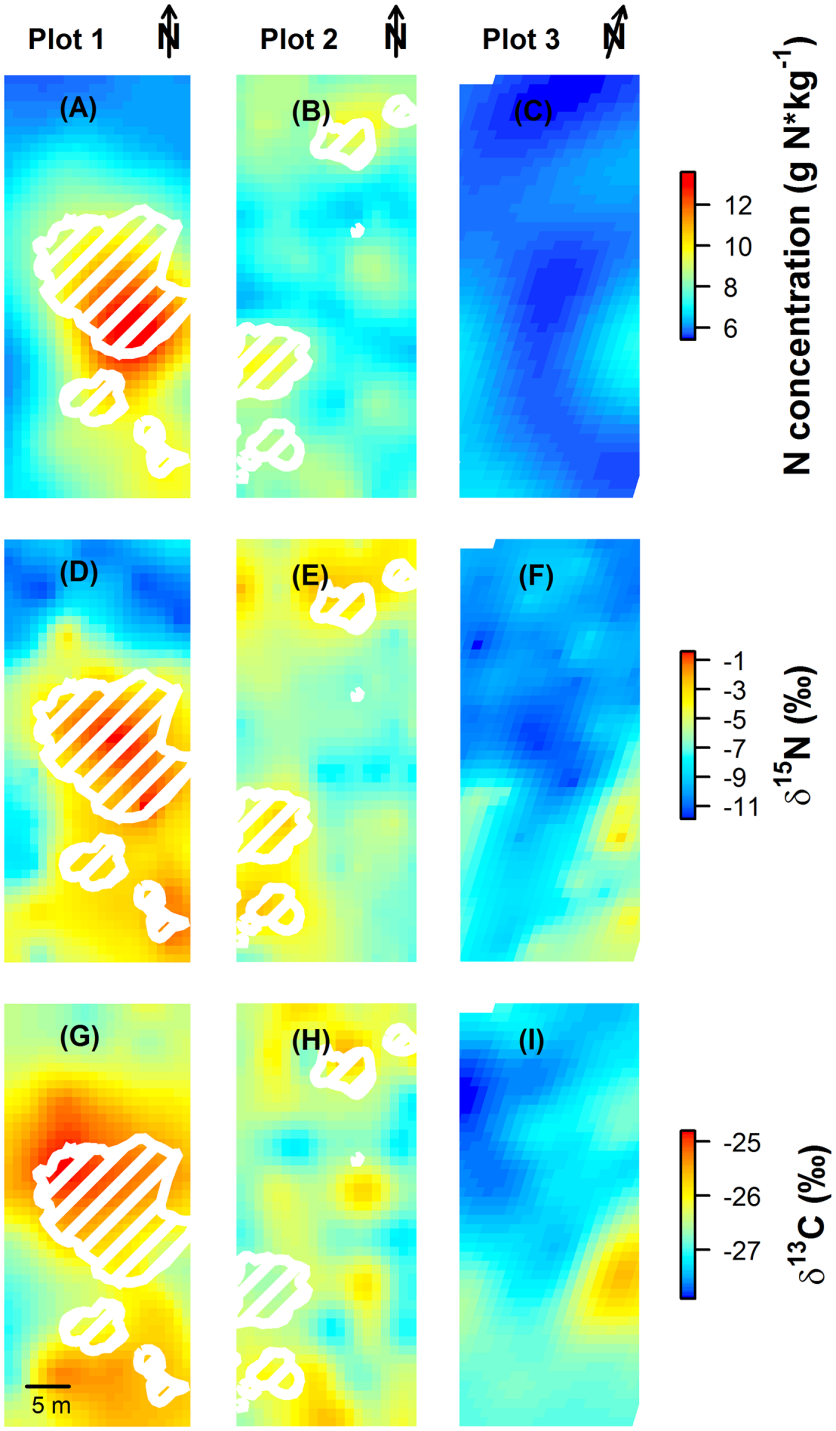
Kriging maps of N concentration (g N*kg^-1^, A-C), δ^15^N (‰, D-F) and δ^13^C (‰, G-I) based on foliage of *Corema album*. Canopies of *Acacia longifolia* in plot 1 and 2 are indicated by white hatched polygons.

The isoscapes revealed distinct spatial patterns in N concentration, δ^15^N and δ^13^C of *C*. *album* foliage (Fig 1). In the invaded plots 1 and 2, N concentration ranged between 5.8–13.5 g N*kg^-1^ and 6.3–9.9 g N*kg^-1^, respectively, and increased considerably when comparing *C*. *album* individuals growing distant and adjacent to *A*. *longifolia* (hatched area, Fig 1A and 1B). These alterations in N concentration corresponded to a more than two-fold increase for plants located close to the invader compared to the uninvaded vegetation at the borders of plot 1, and an enrichment by ca. 30% in plot 2. Similarly, δ^15^N of *C*. *album* became substantially enriched in the vicinity of *A*. *longifolia*, with background values of uninfluenced vegetation of ca. -11‰ (plot 1) and -8‰ (plot 2) increasing to values close to 0‰ (Fig 1D and 1E), even though *C*. *album* itself has no capacity of N_2_-fixation. For δ^13^C, a distinct spatial pattern with values enriched by ca. 2.5‰ for *C*. *album* growing close to the *A*. *longifolia* canopies was evident for plot 1, while for plot 2, pronounced small-scale variation only weakly related to the presence of the invader was observed (Fig 1G and 1H). Noticeably, enrichment in N, δ^15^N and δ^13^C associated with *A*. *longifolia* presence was evident not only in direct neighborhood of the invader, but exceeded the canopy by several meters.

In plot 3, which is not invaded by *A*. *longifolia*, N concentration was extremely low (≤ 7.3 g N*kg^-1^) without much variation throughout the study plot (Fig 1C). δ^15^N showed weak spatial pattern, with enriched values mainly occurring in the southern part of the plot (Fig 1F). However, δ^15^N-enrichment was less in terms of absolute values and spatial extent compared to invaded plots 1 and 2. δ^13^C showed a similar pattern of enrichment, with depleted values in the northern and enriched values in the southern part of the plot and one peak showing maximal values of ca. -25.5‰ (Fig 1I).

**Fig 2:**
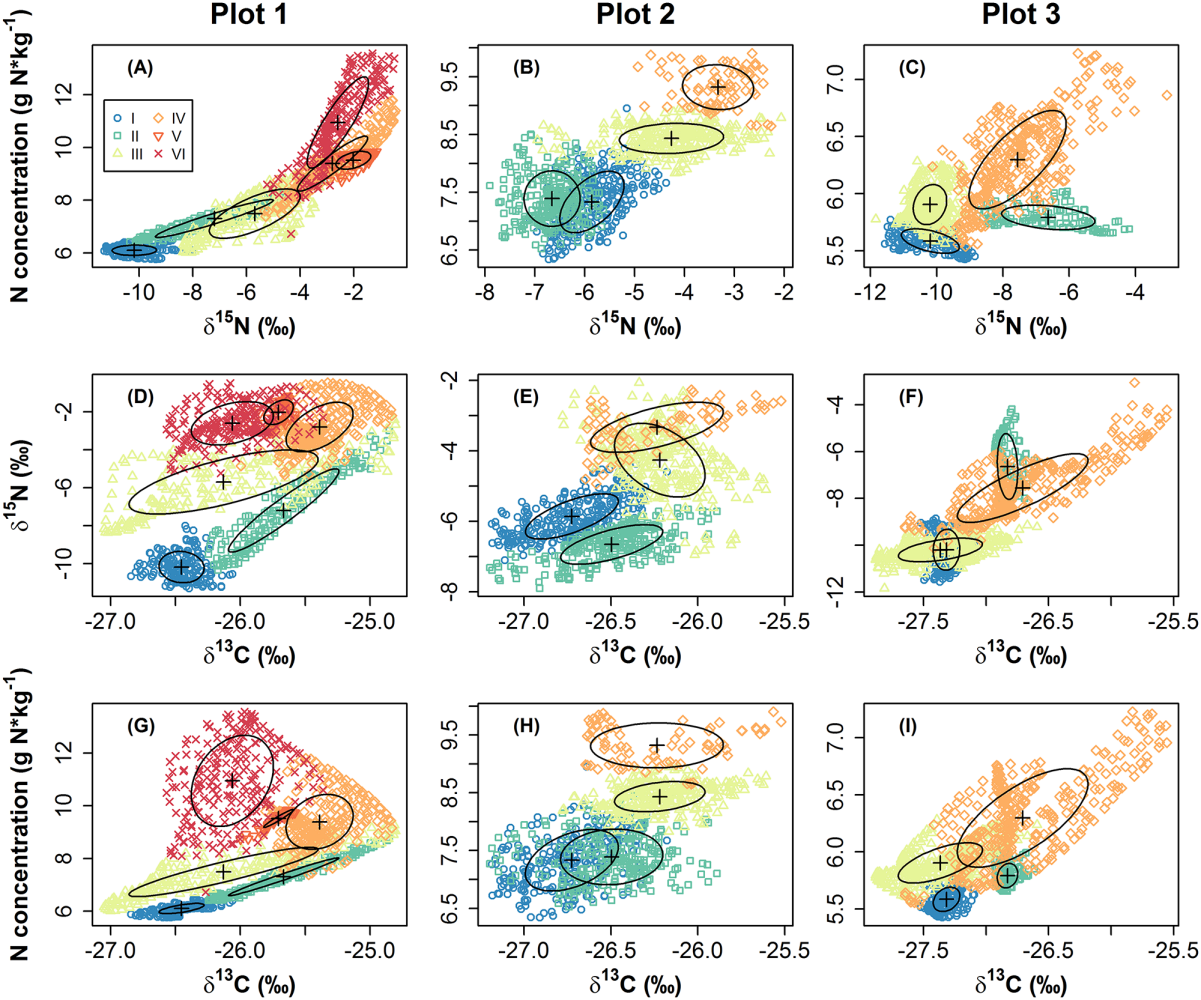
Scatterplots of the variables N concentration (g N*kg^-1^), δ^15^N (‰) and δ^13^C (‰) derived from kriging maps in all combinations for the three study plots. Cluster membership to cluster I to VI (for plot 1) or cluster I to IV (for plots 2 and 3) is indicated by different symbols and coloring, sorted by median N concentration, with blue to red representing low to high N. Ellipses denote the normal-probability contours at probability = 0.5. Please note different scaling of the axes.

Model-based cluster analysis resulted in optimal solutions with six clusters in plot 1 and four clusters in plot 2 and 3 according to the BIC (S1 Fig). Optimal models were ellipsoidal for all three plots, with variable volume, shape and orientation for plot 1, equal volume and shape for plot 2 and equal shape for plot 3 (S1 Fig). Fig 2 shows scatterplots of the three variables used for the cluster analyses, indicating cluster membership of each sample. In all three plots, N concentration and δ^15^N were correlated and clusters were arranged roughly along the concerted increase of these variables, with the exception of cluster II in plot 3 (Fig 2A–2C). Scatterplots of δ^15^N vs. δ^13^C and N concentration vs. δ^13^C similarly showed correlations for plot 3 (Fig 2F and 2I), with cluster II again accommodating a rather outlying group (Fig 2F). Plots 1 and 2 demonstrated a more complex relationship between δ^13^C and δ^15^N: for plot 1, in clusters I, II and III, a positive correlation between δ^15^N and δ^13^C was found, with cluster III being separated from clusters I and II by a higher enrichment in δ^15^N. In contrast, clusters IV, V and VI tended to decrease in δ^13^C with more enriched values in δ^15^N (Fig 2D). The same trend was evident in the scatterplot of N concentration vs. δ^13^C: clusters I, II and III showed increasing values in N concentration with enrichment in δ^13^C, while clusters IV, V and VI on average got depleted in δ^13^C with further increase in N concentration (Fig 2G). A similar pattern, though less pronounced, could be observed in plot 2 (Fig 2E and 2H). While across clusters I, II and III, values tended to correlate positively between δ^13^C and δ^15^N as well as δ^13^C and N concentration, cluster IV, with highest enrichment in δ^15^N and N concentration, did not show further enrichment in δ^13^C.

**Fig 3:**
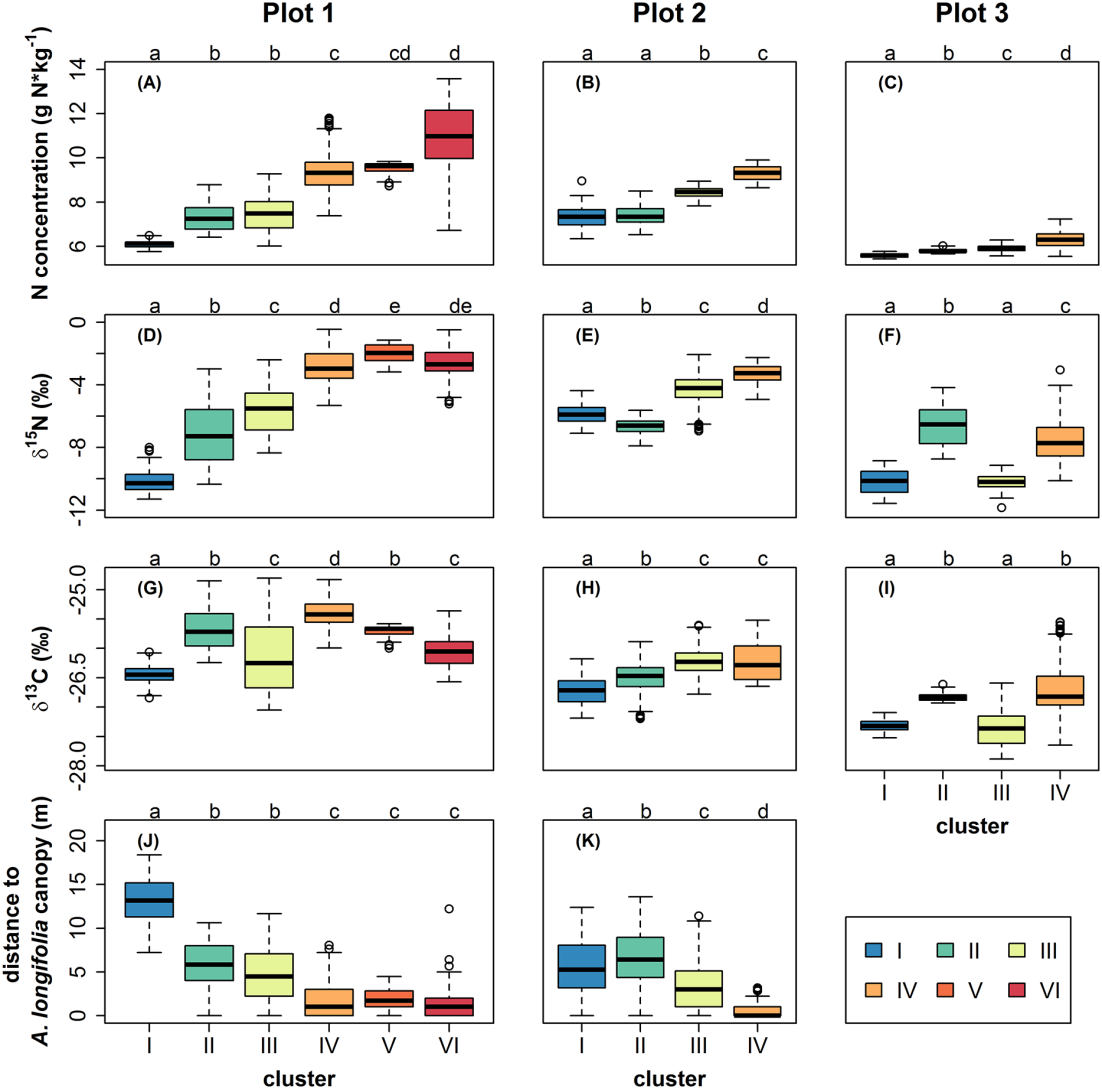
Boxplots illustrating N concentration (g N*kg^-1^, A-C), δ^15^N (‰, D-F) and δ^13^C (‰, G-I) by cluster membership for plots 1, 2 and 3 as well as the distance to the closest *Acacia longifolia* canopy (m, J-K) for invaded plots 1 and 2. Different letters indicate significant differences at *P* < 0.05 (Kruskal-Wallis test, non-parametric multiple comparisons corrected for α-inflation).

Fig 3 shows boxplots of N concentration, δ^15^N and δ^13^C within the individual clusters. For all plots, median N concentration obviously increased with increasing cluster number, as clusters have been ordered by N concentration (Fig 3A–3C). In plot 1, δ^15^N similarly increased from cluster I to V, while cluster VI did not show additional enrichment (Fig 3D). Median δ^13^C increased from cluster I to II and then to IV, but decreased in clusters V and VI. Median δ^13^C of cluster III was intermediate between clusters I and II (Fig 3G). The distance to the closest *A*. *longifolia* canopy of each sample, although it was not a variable in the cluster analysis, mirrored the pattern of medians of δ^15^N, with members of cluster I showing the largest distance, then median distances decreased for clusters II through IV, and members of clusters IV, V and VI were located closest to *A*. *longifolia*.

Patterns in plot 2, while less pronounced, were similar. N concentration increased with cluster number, though it did not differ significantly between clusters I and II (Fig 3B). For δ^15^N, median values were lowest in cluster II and increased in clusters III and IV (Fig 3E). δ^13^C increased from cluster I through III and was not significantly different between clusters III and IV, i.e. δ^13^C did not get further enriched with additional increase in N concentration (Fig 3H). Cluster II was the farthest and cluster IV the closest to the *A*. *longifolia* canopies, and again, the distances accurately mirrored the distribution of δ^15^N (Fig 3K).

In the uninvaded plot 3, i.e. without the influence of *A*. *longifolia*, δ^15^N and δ^13^C increased with increasing N concentration in clusters I, III and IV, however, in group II, relatively low N concentration was associated with the—on average—most enriched δ^15^N and δ^13^C values (Fig 3C, 3F and 3I).

**Fig 4:**
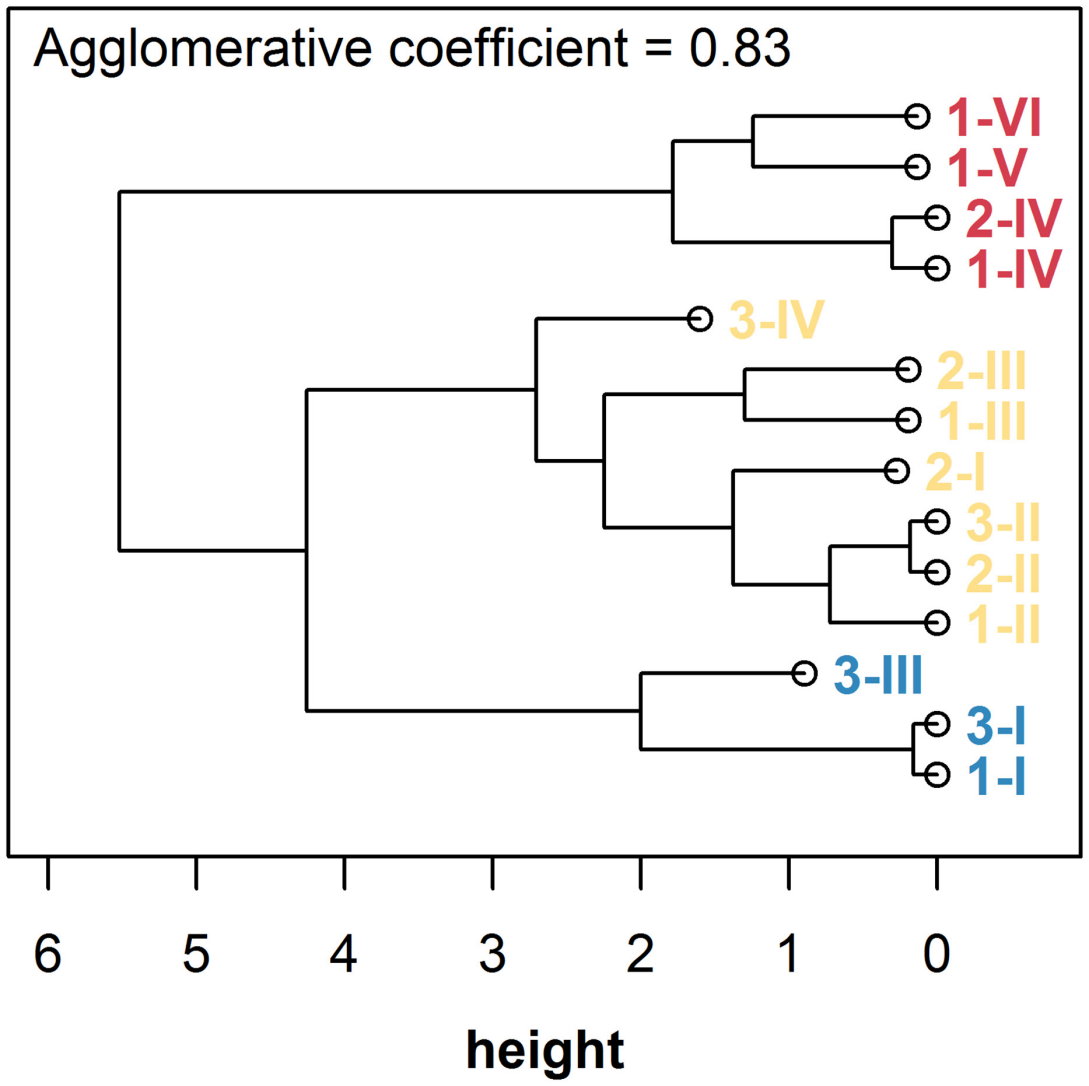
Dendrogram illustrating results of the hierarchical cluster analysis of group medians derived from initial model-based clustering. The optimal solution with k = 3 clusters as identified by highest Silhouette value is indicated by different coloring. Labels specify plot and initial cluster membership in the form *plot*.*cluster* using Arabic and Roman numerals, respectively.

**Fig 5:**
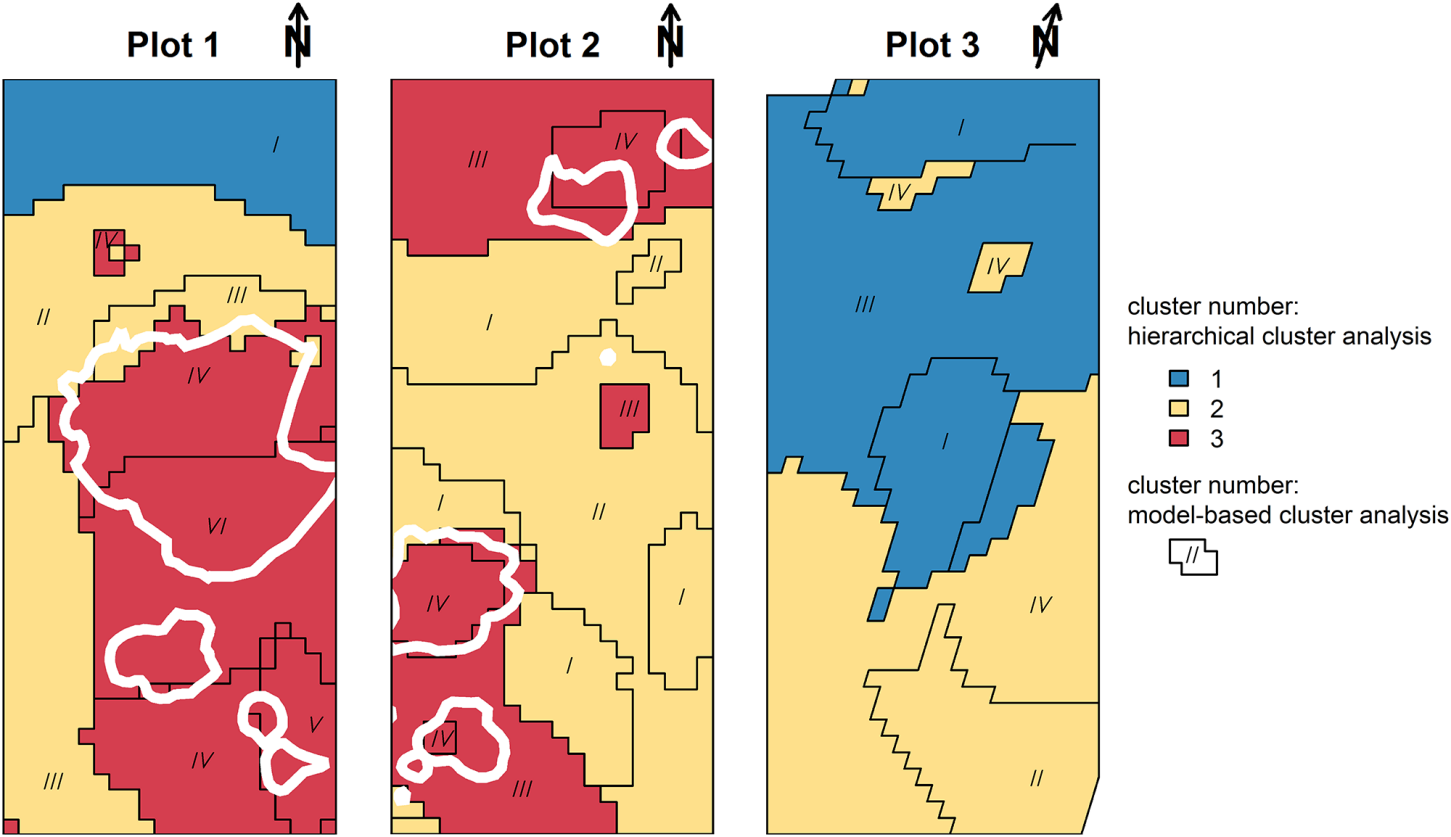
Raster images showing the results of the cluster analysis based on N concentration, δ^15^N and δ^13^C of *Corema album* foliage in the spatial context. Framed areas marked with Roman numerals illustrate membership of cells to clusters yielded by the initial model-based cluster analysis (within plots). Different colors indicate membership to clusters from the final hierarchical cluster analysis (across plots), which was calculated with median values derived from the model-based clustering. Canopies of *Acacia longifolia* in plots 1 and 2 are indicated by white polygons.

Medians of the clusters from the model-based approach were submitted to a hierarchical cluster analysis with the aim to summarize clusters across the three plots. Hierarchical cluster analysis resulted in three final clusters (Fig 4). Generally, the clustering approach yielded spatially homogeneous clusters, though no information on spatial location had been included in the analyses (Fig 5). Final cluster one, which was characterized by low values in all variables and specifically, low δ^15^N with values close to -10‰, was represented at the northern part of plot 1 and covered roughly half of the area of uninvaded plot 3. Final cluster two showed medium δ^15^N and δ^13^C with low to medium N concentration and was present in all three plots, occupying the largest area overall. Final cluster three, with high N concentration and strongly enriched δ^15^N and δ^13^C, only occurred in invaded plots 1 and 2. In these plots, cells assigned to final cluster three spatially corresponded to the locations of *A*. *longifolia* canopies and their surroundings.

## Discussion

Plant invasions are known to alter the structure of plant communities by modifying nutrient flows and water regime [8]. In particular, *Acacia longifolia* populations in south-west Portugal are well known for multiple impacts on plant communities through changes in nutrient, carbon and water cycling [21,22,34]. However, multivariate spatial patterns of its impact have not been analyzed until now. Model-based cluster analysis of three spatially resolved functional tracers, δ^13^C, δ^15^N, and N, was successful in partitioning an area invaded by *A*. *longifolia* into homogeneous clusters on community scale. A subsequent hierarchical cluster analysis then allowed to generalize the results across the individual study plots, yielding three subgroups that can be interpreted in terms of the multivariate impact of the invasive species and allow to distinguish between variation in the measured processes that is inherent to the native system (clusters one and two) and alterations that can be ascribed to the influence of the invader (cluster three, Fig 5). Being based on multiple spatially explicit indictors, the identified zone of influence of *A*. *longifolia* thus comprises integrated information on various processes affected by invasion.

Variation of the measured parameters between and within the uninfluenced clusters depends on intrinsic characteristics of the native system. We found pronounced small scale variation of ca. 2‰ in δ^13^C and up to 8‰ in δ^15^N in final clusters one and two on less than 50 m (Figs 1 and 3; final clusters one and two comprise clusters I-III of plots 1 and 2 and the whole of plot 3; cf. blue and yellow labeling in Figs 4 and 5). This is a large range considering that we followed a standardized sampling procedure within only one target species (cf. e.g. [45–47]). Foliar δ^15^N and δ^13^C both can be expected to vary on small scales with N and water availability, which in turn can be controlled by, e.g., topography, soil texture and plant cover [47,48,49]. Plant δ^15^N depends on the isotopic composition of the N source, species composition, mycorrhizal associations and the relative activity of N transformation processes [16,50,51]. δ^13^C provides an integrated ecological tracer of different structural, functional and phenological attributes [25], which are sensitive to small scale changes in abiotic and biotic environment within a plant community. In particular, plant-plant interactions can directly impact water availability and thereby intrinsic water use efficiency of the interacting individuals in a competitive [33] or facilitative way [52], which will reflect upon the carbon isotope signal. Thus, although mechanistic determinants of variation in plant δ^13^C and δ^15^N have been widely investigated, there is a large gap in data and knowledge on the range and extent of spatial variability within plant communities. Substantial background variation within and between uninvaded clusters one and two, as quantified in this study, thus emphasizes that small scale heterogeneity is highly relevant and has to be taken into account in sampling designs when attempting to use stable isotopes as community-wide tracers [53].

In the invaded final cluster three, all measured parameters were higher, indicating the paramount effects of the invasive species on N budget as well as on photosynthesis and water relations in the indicator species *C*. *album* (Figs 3 and 4). δ^15^N signatures approached the value of atmospheric nitrogen (0‰), which, together with the marked increase in leaf N concentration, suggests uptake and use of N by *C*. *album* that originated from symbiotic N_2_-fixiation (see also [21,22]). Notably, the increase in δ^15^N had a wider range compared to N concentration and accordingly, δ^15^N has previously been suggested as an early indicator for the impact of *A*. *longifolia* [22]. The increase in δ^13^C most likely is the result of two processes both reducing photosynthetic discrimination in *C*. *album* growing close to *A*. *longifolia*: on the one hand, better N nutrition has been shown to increase photosynthetic capacity, leading to a decrease in c_i_/c_a_ (the ratio of internal to ambient CO_2_ partial pressure) and, accordingly, reducing photosynthetic discrimination [54]. On the other hand, as *A*. *longifolia* is a strong competitor for water [33,55], c_i_/c_a_ could also be decreased due to reduced stomatal conductance in response to water stress. Both effects lead to a higher foliar δ^13^C of *C*. *album* in the vicinity of *A*. *longifolia* and indicate higher water use efficiency, but are not necessarily exclusively facilitative, as similarly shown in [33] for a Pine stand invaded by *A*. *longifolia*.

Notably, the native N_2_-fixer *S*. *spectabilis* had no comparable effect on *C*. *album*, as analyzed in detail in [21] and [22]. The higher impact of *A*. *longifolia* compared to *S*. *spectabilis* was mainly attributed to the high growth rates, biomass turnover and litter input of the invader together with its influence on microbial processes as well as on decomposition and turnover rates [21,22].

Cluster analysis provided an objective, unsupervised means to quantify the influence of the invasive *A*. *longifolia* based on a multivariate set of functional tracers and simultaneously enabled to identify the area affected by invasion. Until now, clustering of isoscapes into functionally relevant spatial groups was mainly done for tracing animal movement [28,29], to better understand trophic interactions [30], and to determine origins of biological materials at country level (e.g. [31]). However, detecting spatial patterns with cluster analysis is not trivial because simple cluster algorithms such as hierarchical clustering always produce some sort of clustering even in the absence of any real structure [41]. Ways to improve the analysis are to compare clustering results against a null-model [56], to verify cluster validity or robustness by bootstrapping [57] or to identify the best possible model via model-based clustering [39]. We applied model-based clustering which identified the optimal number of significantly different clusters for each plot. The final step of clustering the median values of the measured parameters per cluster, i.e. δ^13^C, δ^15^N, and N, allowed for comparing all three plots. Hence, model-based cluster analysis proved to be a suitable tool to partition isoscapes into functionally distinct and spatially congruent clusters. In the context of plant invasion, the clusters can then be analyzed with respect to the diverse processes affected by the invader.

Within the affected area, the relationships between the measured processes differed among clusters with different degrees of *A*. *longifolia* influence, as revealed by the initial model-based cluster analysis (Figs 2 and 3). Most intriguingly, while δ^13^C increased with increasing N concentration in the unaffected and marginally influenced clusters, δ^13^C did not increase further with N enrichment in the most strongly influenced clusters. This was particularly true for the heavily invaded plot 1, where median δ^13^C even decreased with higher N concentration in clusters V and VI compared to cluster IV (Figs 2 and 3). Thus, simultaneously analyzing δ^13^C and δ^15^N in a multi-isotope approach revealed a non-linear relationship of N enrichment with water use efficiency that indicated a change in the dominant processes which would not be detectable by analyzing one tracer alone. This pattern might reflect a shift from facilitation by N enrichment in the vicinity of *A*. *longifolia* to competition for resources other than N inside the canopy of the invader, which could have led to a reduction in CO_2_ fixation rates and thus to increasing ratios of c_i_/c_a_. A similar effect was observed in *Acacia*-invaded Pine stand, where indeed intense competition for water can reduce CO_2_ fixation of species in direct neighborhood of *A*. *longifolia* [33]. Also, a spatial change in the balance between facilitation and competition could explain why native plants are eventually outcompeted and replaced by *A*. *longifolia* [21,58,59] despite the beneficial effects on growth rates [21] and WUE_i_ (Figs 1 and 3) which are evident close to the invader in an early stage of invasion. Here, model-based cluster analysis enables to detect these functional shifts within the invaded area.

Although there is emerging awareness that competition and facilitation e.g. in plant-plant, plant-pollinator or plant-microbe interactions could actually have distinct spatial distributions [60–63], the balance between different processes with potentially opposite effects as a function of spatial scale is still rarely addressed in empirical studies. Yet, by not accounting for the high spatial variability and diversity of processes involved, important aspects that drive the outcome of species interactions might be missed. Since plant-plant interactions are truly multivariate and spatially explicit, we posit that to increase our understanding of complex field situations, it will be necessary and consequential to promote the use of innovative multivariate methodologies in the future.

In conclusion, finding homogeneous subgroups in multi-isotope data by means of model-based cluster analysis with spatially resolved measurements of a multivariate set of functional tracers proved very useful in detecting spatial structure in processes affecting plant physiology and performance, including abiotic and biotic factors both, inherent to the native system and imposed by an invasive species. The proposed method can give an objective measure of the spatial extent of influence of plant-plant interactions, potentially providing evidence for shifts between facilitation and competition depending on the spatial scale. Thus, spatial partitioning of isoscapes can help to better understand spatial pattern and interactions in plant communities.

## Supporting Information

S1 Fig Bayesian Information Criterion (BIC) as a function of number of clusters for plots 1–3. Ten different combinations of constraints for multivariate mixture models have been tested: EII = spherical, equal volume; VII = spherical, unequal volume; EEI = diagonal, equal volume and shape; VEI = diagonal, varying volume, equal shape; EVI = diagonal, equal volume, varying shape; VVI = diagonal, varying volume and shape; EEE = ellipsoidal, equal volume, shape, and orientation; EEV = ellipsoidal, equal volume and equal shape; VEV = ellipsoidal, equal shape; VVV = ellipsoidal, varying volume, shape, and orientation.
(PDF)

S1 Table Georeferenced values of δ^15^N (‰), δ^13^C (‰) and N concentration (g N*kg^-1^) used to create isoscapes. (PDF)
