# Supplementary material for: A Spatially Explicit Dual-Isotope Approach to Map Regions of Plant-Plant Interaction after Exotic Plant Invasion
Source: PLoS One. 2016 Jul 27;11(7):e0159403. doi: 10.1371/journal.pone.0159403 (PMC4963087; doi:10.1371/journal.pone.0159403)
Supplement: S1 Fig — Ten different combinations of constraints for multivariate mixture models have been tested: EII = spherical, equal volume; VII = spherical, unequal volume; EEI = diagonal, equal volume and shape; VEI = diagonal, varying volume, equal shape; EVI = diagonal, equal volume, varying shape; VVI = diagonal, varying volume and shape; EEE = ellipsoidal, equal volume, shape, and orientation; EEV = ellipsoidal, equal volume and equal shape; VEV = ellipsoidal, equal shape; VVV = ellipsoidal, varying volume, shape, and orientation. (PDF) [file pone.0159403.s001.pdf]

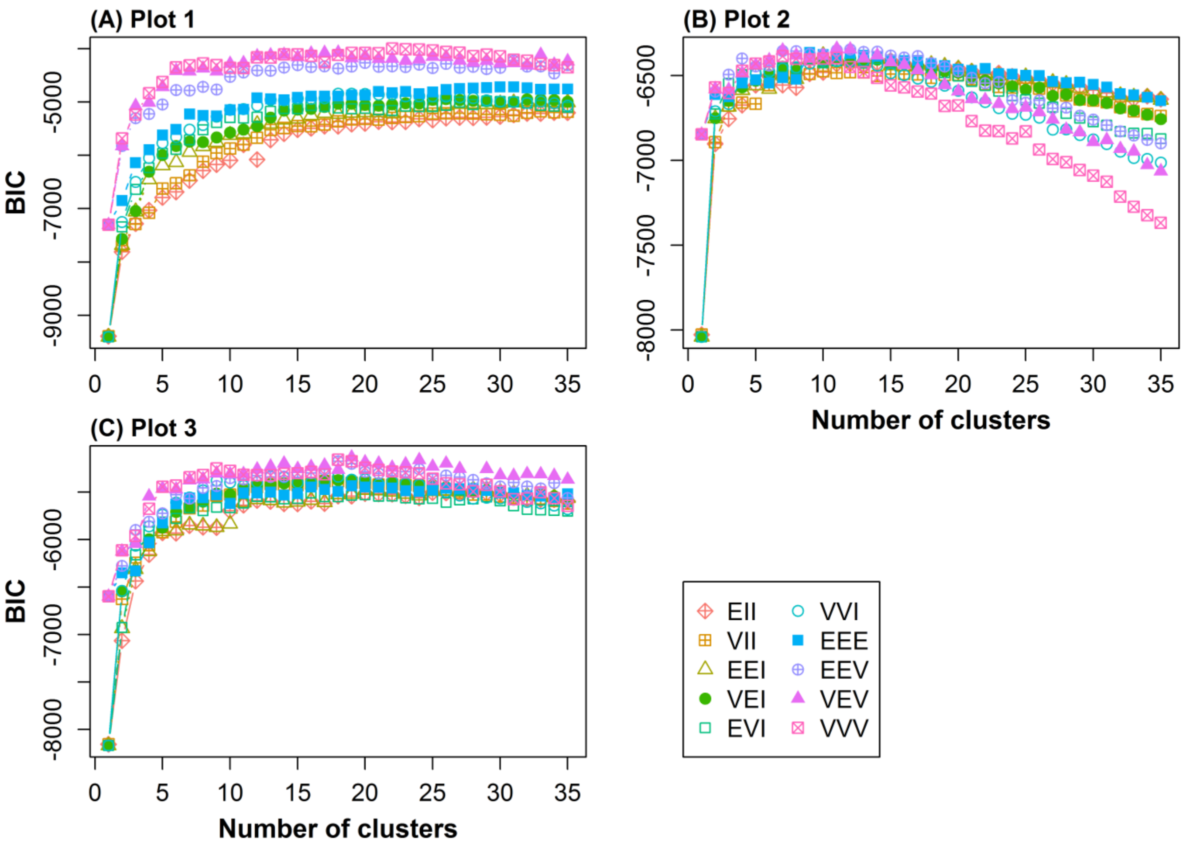

S1 Fig. Bayesian Information Criterion (BIC) as a function of number of clusters for plots 1-3. Ten different combinations of constraints for multivariate mixture models have been tested: EII = spherical, equal volume; VII = spherical, unequal volume; EEI = diagonal, equal volume and shape; VEI = diagonal, varying volume, equal shape; EVI = diagonal, equal volume, varying shape; VVI = diagonal, varying volume and shape; EEE = ellipsoidal, equal volume, shape, and orientation; EEV = ellipsoidal, equal volume and equal shape; VEV = ellipsoidal, equal shape; VVV = ellipsoidal, varying volume, shape, and orientation.
