# Supplementary material for: A Spatially Explicit Dual-Isotope Approach to Map Regions of Plant-Plant Interaction after Exotic Plant Invasion
Source: PLoS One. 2016 Jul 27;11(7):e0159403. doi: 10.1371/journal.pone.0159403 (PMC4963087; doi:10.1371/journal.pone.0159403)
Supplement: S1 Table — (PDF) [file pone.0159403.s002.pdf]

S1 Table. Georeferenced values of δ^{15}N (‰), δ^{13}C (‰) and N concentration (g N*kg^{-1}) used to create isoscapes.

plot.ID	coords.x1	coords.x2	δ^{15}N (‰)	δ^{13}C (‰)	N (g N*kg^{-1})
1	519955.1	4234326.3	-10.9	-26.4	5.8
1	519957.3	4234326.5	-9.9	-26.5	6.2
1	519959.4	4234326.7	-9.9	-26.6	5.4
1	519961.6	4234327	-9.1	-26.4	6.1
1	519963.9	4234327.3	-10.7	-27.6	5.7
1	519966.1	4234327.5	-10.8	-26.7	5.5
1	519968.4	4234327.8	-10.2	-26.7	6.3
1	519970.5	4234328	-9.4	-25.8	6.5
1	519972.7	4234328.2	-8.7	-25.7	5.9
1	519975	4234328.5	-7.6	-26.8	6
1	519955.2	4234321.8	-10.2	-26.4	6.9
1	519957.5	4234322.1	-11.1	-26.6	5.7
1	519959.7	4234322.2	-11.5	-26.2	5.3
1	519961.9	4234322.6	-9.2	-26.6	6.8
1	519964.1	4234323	-9.2	-26.6	6.5
1	519966.3	4234323.4	-9.4	-26.7	6.1
1	519968.5	4234323.6	-9.7	-25.7	6.3
1	519970.7	4234323.6	-11.4	-27.2	6
1	519973	4234323.8	-10.9	-27	6.1
1	519975.3	4234324.1	-10.4	-26.1	6.8
1	519955.4	4234316.5	-9.9	-26.1	5.9
1	519957.6	4234316.7	-8.9	-26.2	7
1	519959.9	4234317	-6.2	-24.9	7.7
1	519962	4234317.2	-3.2	-24.7	8.7
1	519964.2	4234317.6	-7.1	-25.6	7.4
1	519966.5	4234317.8	-7.3	-25.8	6.6
1	519968.6	4234317.9	-8.9	-25.9	6.5
1	519970.8	4234318.2	-9.6	-26.1	6.3
1	519973.2	4234318.4	-9.7	-26.1	6.8
1	519975.6	4234318.7	-11.4	-26.1	5.4
1	519955.5	4234311.3	-5.9	-26	7.4
1	519957.7	4234311.3	-4.9	-25.3	8.7
1	519960	4234311.6	-4.3	-24.8	8
1	519962.2	4234312	-5.2	-24.5	9.2
1	519964.4	4234312.2	-3.5	-25.4	8.5
1	519966.7	4234312.4	-3.1	-25.5	8.7
1	519968.8	4234312.6	-4.7	-25.1	9.6
1	519971	4234313	-5.8	-25.8	7.9
1	519973.2	4234313.1	-5	-25.5	9
1	519975.6	4234313.4	-5.7	-25.8	7.5
1	519955.7	4234305.8	-5.5	-25	8.1
1	519957.9	4234306.1	-4.3	-26.1	8
1	519960.1	4234306.5	-2.3	-24.3	8.7

1	519975.9	4234308	-3.6	-25	7
1	519955.9	4234300.4	-6.7	-26.9	7
1	519958.1	4234300.8	-7.4	-26.2	7.2
1	519960.3	4234301	-3.9	-25.7	8.7
1	519962.5	4234301.3	-3.4	-25	11.5
1	519956.1	4234295.1	-7.9	-26.9	6.3
1	519958.2	4234295.4	-7.5	-26.9	6.1
1	519960.4	4234295.7	-5.9	-26.5	7.8
1	519962.7	4234295.9	-3.4	-26.4	8.8
1	519965	4234296.1	-2.9	-26.8	11.2
1	519967.3	4234296.4	-2.1	-26.2	14
1	519969.4	4234296.8	-2.2	-25.3	13.7
1	519971.6	4234297	0	-26.2	18.2
1	519973.8	4234297	-2.3	-27.2	10.3
1	519976.1	4234297.3	-2.8	-26.7	11.4
1	519956.1	4234289.8	-8.2	-27.1	6.6
1	519958.5	4234290.1	-8	-27	6.2
1	519960.6	4234290.4	-4	-26.6	8
1	519962.8	4234290.5	-3.1	-26.3	7.8
1	519967.3	4234290.8	-3	-26.6	12.5
1	519971.8	4234291.5	-3.3	-25.5	8.4
1	519973.9	4234291.7	-3	-25.2	8.5
1	519976.2	4234292	-3.9	-26.7	6.1
1	519956.4	4234284.5	-4.3	-28.3	5.7
1	519958.5	4234284.6	-3.2	-24.9	8.5
1	519960.7	4234285	-3.9	-24	10.5
1	519963	4234285.2	-3.5	-24.9	9.3
1	519965.2	4234285.6	-3.2	-25.9	8.4
1	519967.5	4234285.7	-2.9	-24.7	8.6
1	519969.7	4234285.8	-2.8	-25.5	8.7
1	519971.9	4234286.1	-2.7	-26.1	9
1	519974	4234286.6	-1.2	-25	12.9
1	519976.3	4234286.9	-2.3	-26.4	7.6
1	519956.3	4234279	-4.4	-25.7	8.3
1	519958.7	4234279.4	-7	-27.1	4.6
1	519960.9	4234279.8	-4.8	-25.7	7.8
1	519963.2	4234280.1	-4.4	-26.2	8.1
1	519965.3	4234280.2	-4.1	-25.6	8.4
1	519967.5	4234280.5	-3.5	-25.3	8.6
1	519969.7	4234280.9	-3.6	-25.7	9.3
1	519972	4234281.1	-2.8	-25.6	10
1	519974.2	4234281.4	-1	-25.9	10.7
1	519976.4	4234281.4	-1.5	-25.3	9.6
2	520022.1	4234259.9	-6.2	-25.9	8.8
2	520024.3	4234259.9	-4.4	-25.3	9.2
2	520026.4	4234260	-4	-26	7.6
2	520028.5	4234260	-3.9	-25.9	8.2

2	520030.7	4234260	-5.6	-26.7	8
2	520032.8	4234259.9	-3.3	-25.8	8.6
2	520035	4234260	-3.2	-26.2	8.5
2	520037.1	4234260	-4	-26.5	8.6
2	520017.9	4234254.9	-1.4	-26.8	7.8
2	520019.9	4234254.8	-4.4	-26.2	9.7
2	520022.2	4234254.8	-6.4	-25.3	8.9
2	520024.2	4234254.9	-2.9	-27.3	7.5
2	520026.4	4234254.8	-1.3	-26.9	8.3
2	520028.5	4234254.9	-2.8	-24.5	11.2
2	520030.7	4234254.8	-2	-25.8	9.4
2	520032.7	4234254.9	-2.9	-25.6	10.5
2	520035	4234255	-3.3	-26.9	8.2
2	520037.1	4234254.8	-5.2	-25.2	8.8
2	520022.1	4234249.8	-5.6	-26	8.2
2	520034.9	4234249.7	-8	-26.9	6.8
2	520037.1	4234249.7	-5.1	-27.8	7.7
2	520017.8	4234244.7	-5.8	-26.6	6
2	520020	4234244.7	-5.2	-27	7.2
2	520022.1	4234244.6	-5.3	-27.2	7.8
2	520024.2	4234244.6	-6.6	-27.8	6.6
2	520030.6	4234244.7	-7.3	-27.1	5.6
2	520032.8	4234244.7	-5.7	-26.4	8.2
2	520034.9	4234244.7	-7	-28	6
2	520017.8	4234239.6	-7.8	-27.1	6.7
2	520020	4234239.6	-6.1	-26.4	7.7
2	520022.1	4234239.6	-6.1	-25.9	7
2	520024.3	4234239.6	-6.1	-26.5	8.6
2	520028.6	4234239.5	-6.2	-26.2	6.5
2	520030.7	4234239.5	-6.2	-25.2	9.3
2	520032.8	4234239.5	-6.6	-25.7	9.2
2	520034.9	4234239.5	-7.8	-26.6	8
2	520037.1	4234239.6	-5.4	-26.8	6.7
2	520017.8	4234234.5	-6.8	-27.4	5.7
2	520019.9	4234234.5	-6.3	-26.6	6.6
2	520022.1	4234234.5	-5.3	-26.3	6.4
2	520024.2	4234234.5	-5.3	-26.2	7.5
2	520026.4	4234234.5	-8.3	-26.5	6.4
2	520028.5	4234234.4	-7.9	-27.2	6.2
2	520030.6	4234234.5	-7.1	-26.7	7.6
2	520032.8	4234234.5	-8.6	-27.2	7.2
2	520034.9	4234234.5	-6.8	-27.4	7.8
2	520037.1	4234234.5	-8.4	-27.5	7.1
2	520017.8	4234229.6	-5	-26.6	10.9
2	520022.1	4234229.5	-1.6	-26.8	9.9
2	520024.2	4234229.4	-2.7	-26.3	9.3
2	520026.4	4234229.4	-6.8	-26.1	8.1

2	520028.5	4234229.4	-6.9	-26.5	6.6
2	520030.6	4234229.3	-5.4	-25.6	7.7
2	520032.8	4234229.3	-5.8	-25.6	6.4
2	520035	4234229.3	-4.5	-27.4	6.4
2	520037.1	4234229.3	-7.1	-27.2	6.1
2	520017.7	4234224.4	-4.4	-26.2	8.8
2	520028.5	4234224.3	-7.6	-28	5.8
2	520030.6	4234224.2	-5.1	-26.2	8.1
2	520032.8	4234224.2	-6.4	-26.2	9
2	520034.9	4234224.3	-5.8	-27.5	8
2	520037.1	4234224.2	-7.4	-27.3	7.5
2	520017.8	4234219.3	-1.9	-26.1	8.5
2	520019.9	4234219.3	-1.6	-25.6	9
2	520026.3	4234219.1	-4.1	-25.2	8.5
2	520028.4	4234219.2	-5.9	-26.7	8.1
2	520030.6	4234219.1	-5.5	-27	7.5
2	520032.8	4234219.1	-7	-26.7	7.2
2	520034.9	4234219.2	-6.7	-26.3	7.6
2	520037.1	4234219.1	-6.9	-26.3	6.8
2	520017.7	4234214.2	-7.1	-26.8	7.2
2	520019.9	4234214.2	-6.7	-26.4	9.1
2	520022.1	4234214.1	-4.9	-25.9	7.7
2	520026.3	4234214.1	-4.6	-25.2	8.4
2	520028.4	4234214.1	-5.3	-25.3	9.5
2	520030.6	4234214	-5.8	-25.9	8.8
2	520032.8	4234214	-6.9	-26.8	6.5
2	520034.9	4234214	-6.1	-25.6	7.1
2	520037	4234214	-7.6	-26.3	8.4
3	519911.4	4234323.6	-9.4	-27.3	5.3
3	519914.9	4234322.2	-10.8	-28.1	5.7
3	519915.7	4234326.4	-10	-26.2	5.7
3	519919	4234324.2	-8.9	-26.3	5.9
3	519918.9	4234327	-9.6	-28	5.2
3	519924.9	4234324	-8.2	-27.3	5.9
3	519926.5	4234326.8	-11.2	-27.9	5.2
3	519929.2	4234325.4	-10.5	-28	5.8
3	519930.9	4234326.4	-10.6	-27	5.9
3	519913.4	4234319.2	-10.5	-28.5	6
3	519917.3	4234317.1	-10.9	-27.9	5
3	519919.4	4234321.8	-9.6	-27	4.4
3	519920.6	4234320.1	-8.3	-28.4	4.9
3	519923.7	4234320.5	-9.4	-27.9	5.4
3	519926.5	4234320.7	-10.4	-27.4	5.8
3	519927.8	4234321.5	-9.9	-27.3	4.6
3	519928.9	4234323.7	-10.8	-26.8	5
3	519932.3	4234321.4	-11.2	-27.6	5.1
3	519914.4	4234314.5	-10	-28.3	5.1

3	519916.5	4234315.4	-12.2	-28	6.1
3	519918.9	4234316.9	-10.4	-27.2	6.6
3	519921.3	4234314.4	-10	-26.8	6.6
3	519922.3	4234316.6	-10.7	-28.3	5.1
3	519925.3	4234314.2	-11	-27.1	5.3
3	519927.7	4234314.3	-8.5	-27.1	6.1
3	519928.5	4234316.3	-8.4	-26.9	8.7
3	519930.3	4234318.7	-11	-27.2	5.7
3	519934.1	4234317.3	-9.6	-26.4	6.9
3	519918	4234307.9	-11.2	-27.7	5.7
3	519919.7	4234309.8	-9.7	-27.8	4.8
3	519919.2	4234311.9	-9.5	-27.9	6.4
3	519923.1	4234308.8	-11	-28.5	5.7
3	519924.1	4234312	-9.9	-26	6.4
3	519927.5	4234309.8	-10.9	-27.5	5.2
3	519928.6	4234311.1	-11.3	-27.9	4.9
3	519930.4	4234309	-10	-27.1	6.3
3	519933.2	4234312.5	-9.4	-25.8	6
3	519934.2	4234312.2	-10.7	-26.9	5.5
3	519918.2	4234303.1	-10.9	-27.4	6.8
3	519920.3	4234305.9	-10.5	-27.9	5.6
3	519924.4	4234305.9	-11.8	-27.6	5.9
3	519926.2	4234304.1	-11.8	-27.7	5.9
3	519927.3	4234306.9	-11.2	-26.4	4.8
3	519930.5	4234304.4	-11.2	-27.2	5.5
3	519932.1	4234306.2	-8.6	-27	5.5
3	519932.9	4234307.1	-8.9	-27.4	5.9
3	519936.7	4234305.6	-7.5	-26.7	5.7
3	519935.4	4234309.5	-10.5	-28.6	5.6
3	519921.1	4234298.8	-5.7	-27	7.7
3	519922.3	4234298.7	-6.1	-27.5	5.9
3	519924.3	4234299.5	-6.4	-27.3	7.2
3	519924.7	4234301.8	-10.2	-27.2	5.6
3	519927.3	4234301.3	-10.4	-26.9	5.4
3	519930.3	4234300.2	-11.3	-27.7	6.2
3	519931.4	4234300.5	-11.9	-28.5	5.3
3	519933.8	4234300.9	-10.2	-26.2	5.4
3	519934.3	4234303.4	-10	-27.5	7.6
3	519938.1	4234300.8	-5.8	-25.9	7.5
3	519936.8	4234303.8	-6.6	-25.9	6.6
3	519921.6	4234296.6	-6.2	-25.7	6
3	519921.2	4234293.7	-6.2	-26.1	6.8
3	519920.5	4234296.7	-6.5	-26.9	6.6
3	519923.4	4234297.1	-7.8	-26.9	6
3	519925.6	4234295	-8.7	-27.7	5.2
3	519927.5	4234294.3	-9.9	-27	5.4
3	519929	4234296.9	-10	-27	5.2

3	519930.8	4234295.4	-8.1	-26.9	6.5
3	519931.2	4234296.6	-9.6	-28.2	6
3	519933.5	4234295.3	-8.2	-25.9	6.1
3	519932	4234298.6	-9.6	-27.2	5.7
3	519934.6	4234299.4	-8	-25.9	5.2
3	519937.6	4234297.6	-3.3	-23.7	8.7
3	519939.8	4234296.2	-6.1	-26	6.6
3	519922.2	4234291.3	-7	-26.3	9
3	519927.9	4234290.3	-8.9	-26.6	5.1
3	519930.8	4234292.2	-8.6	-27.2	5.7
3	519935.3	4234292.3	-7.5	-25.8	6.4
3	519937.9	4234292.2	-6.9	-26.3	7.1
3	519938.2	4234294.1	-1.5	-25.4	6.7
3	519941.4	4234292.5	-7	-25.8	7.9
3	519923.7	4234285.1	-6.8	-27.2	6.5
3	519929.8	4234285.3	-9	-26.3	5.4
3	519931.7	4234288.7	-8.1	-27.5	5.9
3	519934.1	4234286.9	-8.5	-26.5	5.5
3	519935.1	4234289.6	-5.6	-26.5	6
3	519938.7	4234288.1	-8.1	-27.5	5
3	519943.6	4234287.3	-6.6	-26.8	5.4
3	519925.1	4234281.3	-6.8	-26.1	6.6
3	519925.6	4234281.4	-8.5	-27.6	7
3	519928.5	4234282.2	-8.7	-26.9	8.8
3	519929.3	4234282.8	-9.4	-26.8	6.6
3	519932.9	4234282.4	-5.4	-26.9	6.6
3	519934.9	4234284.7	-7	-27	5.9
3	519942	4234285.8	-2.8	-26.5	5.9
